# Predictive value of serological markers and immune indicators combined with early warning scoring system for prognosis in pediatric acute respiratory infections

**DOI:** 10.5937/jomb0-53846

**Published:** 2025-06-13

**Authors:** Peng Zhou, Shanlin Wang, Xinyuan Huang, Caixia Xiang, Xiaoxia Qian, Yaping Shen, Yanyan Zhu

**Affiliations:** 1 Shengzhou People's Hospital (Shengzhou Branch of the First Affiliated Hospital of Zhejiang University School of Medicine, the Shengzhou Hospital of Shaoxing University), Shengzhou, Zhejiang Province, China; 2 Hangzhou Normal University, School of Clinical Medicine, Hangzhou, Zhejiang Province, China; 3 Hangzhou Children's Hospital, Department of Pediatric, Hangzhou, Zhejiang Province, China; 4 Hangzhou Children's Hospital, Department of Rheumatology & Immunology, Hangzhou, Zhejiang Province, China

**Keywords:** acute respiratory infections, pediatric, outcomes, predictors, AVPU scores, serum inflammatory markers, akutne respiratorne infekcije, pedijatrija, ishodi, prediktori, AVPU skorovi, serumski inflamatorni marker

## Abstract

**Background:**

The objective was to evaluate the combined utility of alertness-vigilance-pain-unresponsiveness (AVPU) scoring and serological factors in predicting outcomes for children with acute respiratory infections (ARIs) in the emergency department.

**Methods:**

This retrospective cohort study with a case-control design included 100 children with ARIs admitted to a pediatric department from May 2022 to May 2024. Patients were divided into the good prognosis group (GPG) and the poor prognosis group (PPG) based on their outcomes. Clinical data, vital signs, alertness-vigilance-pain-unresponsiveness (AVPU) scores, serum inflammatory markers (SIMs), immunoglobulin levels, and immune cell counts were compared between the two groups.

**Results:**

The GPG had significantly lower WBC, CRP, IL-6, and PCT levels than the PPG. AVPU scores were substantially lower in the PPG. Pearson correlation analysis revealed no notable correlation between AVPU scores and SIMs. Receiver operating characteristic (ROC) curve analysis showed that AVPU scores had higher sensitivity and specificity for predicting unfavourable outcomes than SIMs.

**Conclusions:**

AVPU scores and SIMs are valuable predictors of unfavourable outcomes in pediatric ARIs. Combined testing of AVPU scores and SIMs may improve predictive performance. These findings can inform early identification and timely intervention for children with ARIs at risk of unfavourable outcomes.

## Abstract (sr)

**Uvod:**

Cilj je bio da se proceni kombinovana korist skora budnost-opreznost-bol-odsustvo odgovora (AVPU) i seroloških faktora u predikciji ishoda kod dece sa akutnim respiratornim infekcijama (ARI) u hitnoj službi.

**Metode:**

Ova retrospektivna kohortna studija sa dizajnom studije slučaja i kontrole je obuhvatila 100 dece sa akutnim respiratornim infekcijama (ARI) primljenih na pedijatrijsko odeljenje od maja 2022. do maja 2024. godine. Pacijenti su podeljeni u grupu sa dobrom prognozom (GPG) i grupu sa lošom prognozom (PPG) na osnovu ishoda. Izme|u ove dve grupe su upore|eni klinički podaci, vitalni znaci, skorovi budnost-opreznost-bol-odsustvo odgovora (AVPU), serumski inflamatorni markeri (SIM), nivoi imunoglobulina i broj imunskih }elija.

**Rezultati:**

Grupa sa dobrom prognozom (GPG) je imala značajno niže vrednosti leukocita (WBC), C-reaktivnog proteina (CRP), interleukina-6 (IL-6) i prokalcitonina (PCT) u pore|enju sa grupom sa lo{om prognozom (PPG). AVPU skorovi su bili značajno niži u grupi sa lošom prognozom. Pirsonova korelaciona analiza nije pokazala značajnu korelaciju između AVPU skorova i serumskih inflamatornih markera (SIM). Analiza ROC krive je pokazala da AVPU skorovi imaju veću senzitivnost i specifičnost u predikciji nepovoljnih ishoda u poređenju sa SIM. Kombinovano testiranje AVPU skorova i SIM je pokazalo ve}u senzitivnost i specifičnost nego pojedinačni indikatori.

**Zaključak:**

AVPU skorovi i serumski inflamatorni markeri (SIM) predstavljaju značajne prediktore nepovoljnih ishoda kod akutnih respiratornih infekcija (ARI) kod dece. Kombinovano testiranje AVPU skorova i SIM može da poboljša prediktivnu efikasnost. Ova otkrića mogu da doprinesu ranom prepoznavanju i pravovremenoj intervenciji kod dece sa ARI koja su u riziku od nepovoljnog ishoda.

## Introduction

In contemporary medical practice, managing pediatric emergency acute respiratory infection (ARI) is a significant and complex issue. ARI encompasses a range of conditions, from minor colds to severe pneumonia and is one of the most common health concerns in children [1] [2]. Although most cases present with mild symptoms, some can rapidly deteriorate and pose a threat to the child’s life, making early identification, treatment, and prognostic assessment particularly critical [3]. Early warning scoring systems in pediatric emergency settings provide physicians with effective tools to assess the severity of a child’s condition and predict outcomes. These scoring systems typically incorporate vital signs such as temperature (Temp), respiratory rate (RR), heart rate (HR), and oxygen saturation (SpO_2_), along with laboratory test results, including white blood cell (WBC) count and C-reactive protein (CRP) as serum inflammatory markers (SIMs) [4]. By analyzing these indicators comprehensively, physicians can quickly assess changes in the child’s condition, adjust treatment plans promptly, and mitigate the risk of adverse outcomes [5].

Simultaneously, the role of serological factors in the predictive assessment of pediatric ARI has garnered increasing attention. Elevated WBC counts, and the release of specific cytokines reflect the degree of the immune response. These SIMs help gauge the severity of the condition and guide the formulation and adjustment of treatment strategies [6]. For instance, some studies indicated that IL-6 levels are closely related to ARI severity and treatment response, making it a crucial prognostic marker. Early warning scoring systems and serological factors can enhance pediatric ARI cases’ management and predictive accuracy in emergency settings [7] [8]. Early identification of high-risk patients allows for prompt and aggressive treatment measures, reducing the risk of unnecessary hospitalizations and complications.

Moreover, personalized treatment strategies can significantly improve affected children’s condition and quality of life [9] [10]. Nevertheless, despite their theoretical and preliminary practical potential, several challenges and limitations persist in real-world applications. First, epidemiological and immunological characteristics of ARI may vary across different institutions and regions, necessitating adjustments and validation according to specific circumstances [11] [12] [13]. Additionally, the standardization and uniformity of scoring systems and serological factors require further refinement to ensure their stability and reliability across diverse clinical settings [14].

This work explored the potential value and clinical adoption prospects of combining early warning scoring systems with serological factors in the prognostic assessment of pediatric emergency ARIs. By synthesizing existing literature and recent research findings, it investigated these methods’ practical effects and challenges in the early identification of high-risk children, guiding personalized treatment, and improving outcomes. The goal was to provide theoretical support and clinical guidance for optimizing management strategies for pediatric emergency respiratory infections.

## Materials and methods

This study is a retrospective cohort study with a case-control design performed on children with ARIs admitted to the pediatric department of Shengzhou People’s Hospital (Shengzhou Branch of the First Affiliated Hospital of Zhejiang University School of Medicine, the Shengzhou Hospital of Shaoxing University) from May 2022 to May 2024. The study subjects, with the consent of their family members, agreed to sign an informed consent form. The hospital’s ethics committee approved this work’s implementation with code No. 2022KY1323 from the Medical and Health Science and Technology Project of Zhejiang Province.

### Study population

The patients were identified through a review of electronic medical records. They were included if they had a confirmed diagnosis of ARI, defined as a respiratory infection with clinical symptoms and radiographic evidence of pneumonia or bronchiolitis.

Based on Zhang et al. (3) study, which showed that a WBC count >15×10^9^/L is an independent risk factor for poor prognosis in children with severe infections (OR: 1.725), a sample size calculation was performed. Assuming a moderate effect size and a prevalence of WBC >15×10^9^/L around 35%, a sample size of approximately 100 was estimated to achieve 80% power and a 5% significance level.

Inclusion criteria: i. Onset and worsening of symptoms within one week; ii. Complete clinical, laboratory, and imaging data; iii. Meeting the diagnostic criteria for pediatric respiratory infections; iv. Children who have not yet received treatment.

Exclusion criteria: i. Severe asphyxia; ii. Severe intracranial hemorrhage; iii. Inability to actively cooperate with the entire examination process; iv. Loss of important research data or key information; v. Coexisting chronic active pulmonary diseases such as tuberculosis.

The study population was divided into two groups based on their outcomes. Based on their outcomes, the children were rolled into the good prognosis group (GPG, n=79) and poor prognosis group (PPG, n=21):

- Good Prognosis Group (GPG): patients who were discharged from the hospital without any complications and had a length of stay 3 days
- Poor Prognosis Group (PPG): patients who required ICU admission, had a length of stay >3 days, or died during hospitalization

### Hematological examination methodologies

Serum immune and inflammatory markers were measured employing ELISA kits (Jiangsu isite Biotechnology Co., Ltd., China). The procedure was as follows. Blood samples were collected from the children’s peripheral blood and centrifuged to obtain serum. The serum was diluted with an appropriate dilution buffer to ensure it fell within the detection range of the kit. Anti-IL-6 or anti-IgE antibodies (primary antibodies) were immobilized at the bottom of the wells on an ELISA plate by applying 100 μL of antibody solution per well and incubating overnight at 4°C or for 1 hour at 37°C. Following this, 100 μL of diluted serum samples were applied to the respective wells and incubated at 25°C for 1–2 hours. Each well was rinsed 3–5 times with 200 μL of PBS-T buffer. Subsequently, 100 μL of enzyme-conjugated secondary antibodies (e.g., anti-human IL-6-HRP or anti-human IgE-HRP) were applied and incubated at 25°C for 30–60 minutes, followed by washing 3–5 times. Afterwards, 100 μL of 3,3’,5,5’-Tetra methylbenzidine (TMB) substrate solution was applied and incubated at 25°C for 15–30 minutes without light. Then, 100 μL of stop solution (e.g., 2 mol/L sulfuric acid) was applied. Finally, absorbance was measured at 450 nm via an ELISA reader, and the concentrations of IL-6 and IgE antibodies in the samples were determined by comparing them with a standard curve.

### Data collection

(1) Clinical data of the children, including age, sex, height, weight, medical history, length of hospital stay, and main symptoms at admission, were collected.

(2) Common physiological indicators at admission were recorded, including HR, RR, blood pressure (BP), body Temp (Temp), and SpO_2_.

(3) The alertness-vigilance-pain-unresponsiveness (AVPU) score at admission was collected. The AVPU score was adopted for rapid assessment of the child’s consciousness state, with the following scoring criteria: A (Alert) indicates the child is fully awake, aware, able to interact with the environment, answer questions, and follow commands, scoring 4 points. V (Voice) indicates the child responds to verbal stimuli but may not be fully alert, showing responses such as eye-opening, head turning towards the sound, or making sounds, scoring 3 points. P (Pain) indicates the child responds to pain stimuli but does not respond spontaneously, with signs such as avoidance movements, eye-opening, or body movement, scoring 2 points. U (Unresponsive) indicates the child shows no response, including to pain stimuli, with no eye opening or body movement, scoring 1 point. A higher AVPU score reflects a better level of consciousness, indicating an increased responsiveness to stimuli.

(4) SIMs at admission were collected, including WBC count, CRP, IL-6, and procalcitonin (PCT).

(5) Immune cell factors at admission were collected, including IgA, IgG, IgM, CD3+ T cells, CD4+ T cells, CD8+ T cells, CD19+ T cells, and NK cells.

### Statistical methods

Data were analyzed utilizing *SPSS 22.0*. Continuous variables (normal distribution) were recorded as mean ± standard deviation, while categorical variables were recorded as frequencies and percentages (%). The Mann-Whitney U test was applied for continuous variables not conforming to a normal distribution, whereas normally distributed continuous variables were compared via a one-way analysis of variance. Categorical variables were compared by X^2^ test. Pearson correlation analysis assessed the relationship between AVPU scores, SIMs, and prognosis. The diagnostic performance of factors for pediatric emergency ARIs was evaluated using ROC curves, with AUC calculated. Statistically, a two-tailed level of *P*<0.05 was significant.

## Results

**Figure 1:**
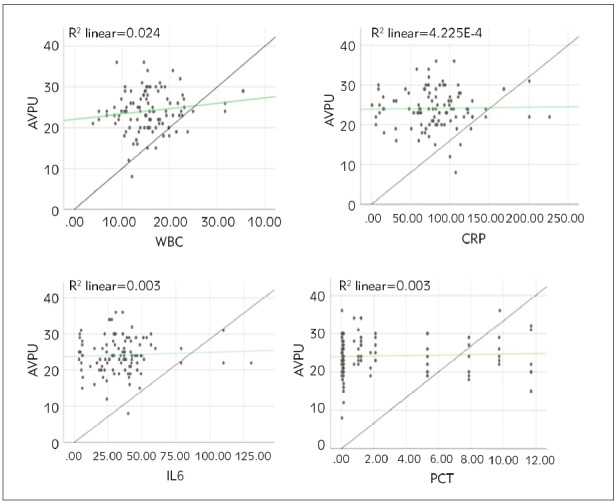
Correlation analysis between AVPU score and SIMs in pediatric patients. (A is the correlation between AVPU score and WBC; B is the correlation between AVPU score and CRP; C is the correlation between AVPU score and IL-6; D is the correlation between AVPU score and PCT).

The basic information of the children is illustrated in Figure 1. In the GPG, there were 46 males and 33 females, with mean age of 5.07±2.91 years, average weight of 20.98±7.84 kg, and average height of 107.63±19.16 cm. This group had 12 cases with a history of previous illness. In the PPG, there were 13 males and 8 females, with an average age of 5.43±3.40 years, a mean weight of 21.5±9.18 kg, and a mean height of 111±24.66 cm. This group had 3 cases with a history of previous illness. The differences in gender ratio, age, weight, height, and the number of prior illness cases between the favourable and PPGs were neglectable (*P*>0.05). Vital signs are shown in Table 1. The HR, RR, Temp, systolic BP, diastolic BP, and SpO_2_ in the GPG differed slightly from those in the PPG (*P*>0.05). The AVPU scores are shown in Table 1. The AVPU scores at admission in the GPG were markedly inferior to the PPG, with the difference being drastic (*P*<0.05).

**Table 1:** Detailed information on single nucleotide polymorphisms (SNPs) related to leakage factors and outcome factors.

Characteristics	GPG (n=79)	PPG (n=21)	P-value
Basic Information			
Male/Female	46/33	13/8	0.43
Age (years)	5.07±2.91	5.43±3.40	0.67
Weight (kg)	20.98±7.84	21.5±9.18	0.81
Height (cm)	107.63±19.16	111±24.66	0.62
Previous illness cases	12	3	0.21
Vital Signs			
Heart Rate (bpm)	120±20	130±25	0.12
Respiratory Rate (bpm)	25±5	28±6	0.09
Temperature (°C)	37.5±1.0	38.0±1.2	0.11
Systolic BP (mmHg)	90±10	95±12	0.15
Diastolic BP (mmHg)	60±8	65±10	0.23
SpO_2_ (%)	95±5	92±6	0.08
AVPU Scores			
AVPU score	8.2±1.5	10.5±2.0	0.001

The comparison of serum inflammatory markers, immunoglobulin levels, and immune cell counts between the Good Prognosis Group (GPG) and Poor Prognosis Group (PPG) is presented in Table 2. The GPG had significantly lower levels of WBC (12.45± 2.13 vs 18.23±3.52, p=0.0012), CRP (20.82± 5.21 vs 35.14±7.13, p=0.0025), IL-6 (40.25±10.57 vs 60.15±15.19, p=0.0043), and PCT (0.52±0.12 vs 1.23±0.34, p=0.0011) compared to the PPG. However, the levels of IgA (1.53± 0.31 vs 1.73±0.41), IgG (8.52±1.23 vs 9.15± 1.51), and IgM (2.15±0.53 vs 2.43±0.63) did not differ significantly between the two groups. Similarly, the immune cell counts, including CD3+ cells (1.23±0.32 vs 1.45±0.42), CD4+ cells (0.83± 0.22 vs 0.94±0.31), CD8+ cells (0.43±0.12 vs 0.52± 0.21), CD19+ cells (0.23±0.11 vs 0.31±0.14), and NK cells (0.14±0.11 vs 0.22±0.13) were not significantly different between the GPG and PPG.

**Table 2:** Comparison of Serum Inflammatory Markers, Immunoglobulin Levels, and Immune Cell Counts between Good Prognosis Group (GPG) and Poor Prognosis Group (PPG).

Characteristics	GPG (n=79)	PPG (n=21)	P-value
Serum Inflammatory Markers			
WBC (×10^9^/L)	12.45±2.13	18.23±3.52	0.0012
CRP (mg/L)	20.82±5.21	35.14±7.13	0.0025
IL-6 (pg/mL)	40.25±10.57	60.15±15.19	0.0043
PCT (ng/mL)	0.52±0.12	1.23±0.34	0.0011
Immunoglobulin Levels			
IgA (mg/mL)	1.53±0.31	1.73±0.41	0.235
IgG (mg/mL)	8.52±1.23	9.15±1.51	0.313
IgM (mg/mL)	2.15±0.53	2.43±0.63	0.413
Immune Cell Counts			
CD3+ cells (×10^9^/L)	1.23±0.32	1.45±0.42	0.181
CD4+ cells (×10^9^/L)	0.83±0.22	0.94±0.31	0.294
CD8+ cells (×10^9^/L)	0.43±0.12	0.52±0.21	0.354
CD19+ cells (×10^9^/L)	0.23±0.11	0.31±0.14	0.224
NK cells (×10^9^/L)	0.14±0.11	0.22±0.13	0.193

### The correlation between AVPU score and SIMs in pediatric patients

Based on the intergroup data comparison results, Pearson correlation analysis assessed the relationship between AVPU scores and SIMs (Figure 1). The analysis revealed that AVPU scores were not notably correlated with the SIMs WBC, CRP, IL-6, and PCT (*P*>0.05).

### ROC curve of AVPU score combined with SIMs in pediatric patients

**Figure 2:**
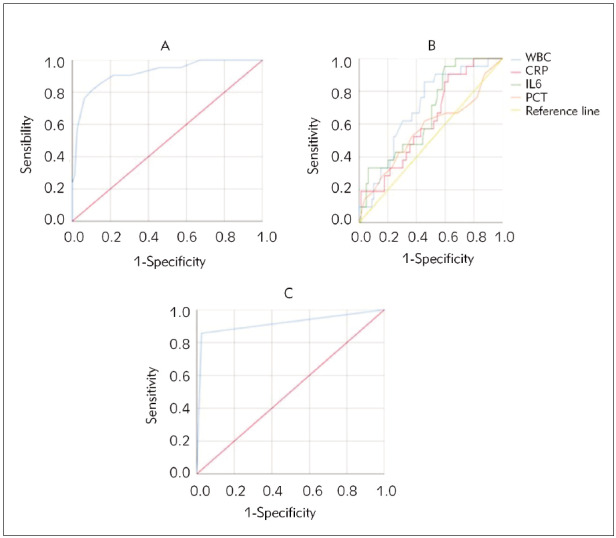
ROC curve of AVPU score combined with SIMs in pediatric patients. (A is the ROC curve of AVPU score; B is the ROC curve of SIMs; C is the ROC curve of combined indicators).

ROC curves were generated to evaluate the predictive performance of AVPU scores and SIMs (Figure 2). The results indicated that AVPU scores had a sensitivity (Sen) of 91.14%, specificity (Spe) of 80.95%, and an AUC of 0.920 for predicting unfavourable outcomes. WBC count had a Sen of 81.01%, Spe of 71.43%, and an AUC of 0.698. CRP showed a Sen of 64.56%, Spe of 52.38%, and an AUC of 0.620. IL-6 had a Sen of 74.68%, Spe of 61.90%, and an AUC of 0.668. PCT demonstrated a Sen of 59.49%, Spe of 66.67%, and an AUC of 0.568. Combined testing yielded a Sen of 97.47%, Spe of 85.71%, and an AUC of 0.916 for predicting unfavourable outcomes.

**Figure 3:**
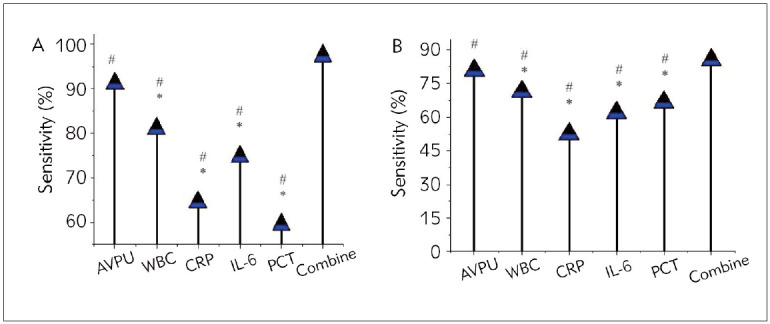
Comparison of diagnostic Sen and Spe of AVPU score combined with SIMs in pediatric patients. (A is Sen; B is Spe) Note: * indicates a great difference in AVPU scores (P<0.05); # implies a notable difference (P<0.05) versus combined detection.

In Figure 3, among single indicators, AVPU scores exhibited higher Sen and Spe for predicting unfavourable outcomes versus SIMs (WBC, CRP, IL-6, PCT) (*P*<0.05). Additionally, combined detection demonstrated drastically higher Sen and Spe for predicting unfavourable outcomes versus individual indicators, with marked differences (*P*<0.05).

## Discussion

This study selected 100 children with emergency ARIs to further evaluate the prognostic value. The children were categorized into GPG (79 cases) and PPG (21 cases). Comparison of baseline data and vital signs revealed neglectable differences in gender ratio, age, weight, height, number of previous illness cases, HR, RR, body Temp, systolic BP, diastolic BP, and SpO_2_ between the two groups (*P*>0.05). This provides a reliable basis for subsequent analyses of AVPU scores, SIMs, and immune indicators between the groups. The AVPU score is a simple tool for assessing consciousness levels and is commonly utilized in emergency and acute care settings. In children with ARIs, changes in consciousness may indicate the severity of the illness or the presence of complications. The AVPU score aids in rapidly assessing a child’s consciousness state, facilitating the early detection of potentially severe conditions [15]. This study found that the AVPU scores at admission were lower in the GPG than the PPG (*P*<0.05), suggesting that children with an unfavourable prognosis had poorer consciousness levels. This indicates a more severe condition or possible central nervous system involvement, with lower AVPU scores potentially correlating with worse outcomes. Li et al. [16] analyzed clinical data from 118 patients with acute community- acquired lower respiratory tract infections. They found that patients with complications had higher WBC counts, PCT, CRP, and erythrocyte sedimentation rates than those without complications. Similarly, it was observed that SIMs WBC, CRP, IL-6, and PCT at admission were drastically lower in the GPG than the PPG (*P*<0.05).

Our study suggests that AVPU scores and SIMs may be useful in predicting unfavourable outcomes in children with ARIs, while Rees et al.’s [17] study raises concerns about the external validity of existing clinical prediction rules (RISC, RISC-Malawi, and PERCH) for identifying children at risk of hospitalized pneumonia-related mortality.

Fernandes et al.’s [18] study identifies obesity, hypoxia on admission, lower absolute lymphocyte count, and more significant C-reactive protein as predictors of severe SARS-CoV-2 disease manifestations, while our study identifies AVPU scores and SIMs (higher WBC, CRP, IL-6, and PCT) as predictors of unfavourable outcomes. The apparent discrepancy between studies may be attributed to several factors. Firstly, the two studies evaluate different outcomes: Fernandes et al.’s study focuses on MIS-C, a distinct clinical entity characterized by a systemic inflammatory response, whereas our study assesses the severity of ARIs in general. Secondly, the immune response to SARS-CoV-2 infection, as studied by Fernandes et al., may differ from the immune response to other ARIs. Lymphopenia, or low lymphocyte count, is a common feature of SARS-CoV-2 infection, particularly in severe cases, whereas a high WBC count may be more indicative of a bacterial or viral infection other than SARS-CoV-2.

The study on the diagnostic performance of calprotectin in respiratory tract infections [19] shares similarities with our study in that both focus on respiratory tract infections and aim to identify biomarkers that can help distinguish between bacterial and viral infections. However, differences exist in the biomarkers evaluated, with your study assessing AVPU scores, WBC, CRP, IL-6, and PCT, whereas the calprotectin study evaluates calprotectin, HBP, and PCT. In the study [19], PCT levels were significantly higher in patients with bacterial pneumonia than those with viral infections.

Procalcitonin levels increase in children with bacterial pneumonia because procalcitonin is a biomarker that rises in response to serious bacterial infections. In patients with viral infections, which do not respond to antibiotics, procalcitonin levels are suppressed [19] [20]. The pathophysiology of procalcitonin increase in children with pneumonia is complex and not fully understood. However, studies have shown that procalcitonin is upregulated in bacterial infections by releasing tumour necrosis factor and interleukin-1 and interleukin-6 and inhibited in viral infections through interferon [21] [22] [23].

### Study limitations

The study has several limitations, including a retrospective design, a small sample size of 100 patients, and a limited population from a single hospital in China, which may not represent other populations or settings. Additionally, the study does not control for potential confounding variables, such as underlying medical conditions, comorbidities, or treatment differences, which may affect the results. The follow-up period is also limited to up to 3 days, which may not be sufficient to capture the full range of outcomes or assess the biomarkers’ long-term predictive value. Furthermore, the study does not validate the biomarkers used, and the findings may not be generalizable to other populations or settings, such as community-based settings or developing countries. Moreover, the study does not consider the viral or bacterial causes of ARIs, which may affect the predictive value of the biomarkers and does not account for treatment differences between the good prognosis group and the poor prognosis group, which may impact outcomes.

## Conclusion

This study found that AVPU scoring is a valuable tool for predicting the prognosis of children with emergency acute respiratory infections and that combining it with serum inflammatory markers (SIMs) can improve the accuracy of prognosis evaluation. However, the study’s small sample size and limited data collection period are limitations that need to be addressed in future research to validate and generalize the findings.

## Dodatak

### Funding

The present study was funded by the Medical and Health Science and Technology Project of Zhejiang Province (No. 2022KY1323).

### Conflict of interest statement

All the authors declare that they have no conflict of interest in this work.
